# Sorting the Healthy Diet Signal from the Social Media Expert Noise: Preliminary Evidence from the Healthy Diet Discourse on Twitter

**DOI:** 10.3390/ijerph17228557

**Published:** 2020-11-18

**Authors:** Theo Lynn, Pierangelo Rosati, Guto Leoni Santos, Patricia Takako Endo

**Affiliations:** 1 Irish Institute of Digital Business, Dublin City University, Dublin, Ireland; theo.lynn@dcu.ie; 2 Centro de Informática, Universidade Federal de Pernambuco, Recife 52071-030, Brazil; gls4@cin.ufpe.br; 3 Programa de Pós-Graduação em Engenharia da Computação, Universidade de Pernambuco, Recife 50100-010, Brazil; patricia.endo@upe.br

**Keywords:** healthy diet, diet, Twitter, obesity, nutrition, social media, public health communications, social influencers, influence marketing

## Abstract

Over 2.8 million people die each year from being overweight or obese, a largely preventable disease. Social media has fundamentally changed the way we communicate, collaborate, consume, and create content. The ease with which content can be shared has resulted in a rapid increase in the number of individuals or organisations that seek to influence opinion and the volume of content that they generate. The nutrition and diet domain is not immune to this phenomenon. Unfortunately, from a public health perspective, many of these ‘influencers’ may be poorly qualified in order to provide nutritional or dietary guidance, and advice given may be without accepted scientific evidence and contrary to public health policy. In this preliminary study, we analyse the ‘healthy diet’ discourse on Twitter. While using a multi-component analytical approach, we analyse more than 1.2 million English language tweets over a 16-month period in order to identify and characterise the influential actors and discover topics of interest in the discourse. Our analysis suggests that the discourse is dominated by non-health professionals. There is widespread use of bots that pollute the discourse and seek to create a false equivalence on the efficacy of a particular nutritional strategy or diet. Topic modelling suggests a significant focus on diet, nutrition, exercise, weight, disease, and quality of life. Public health policy makers and professional nutritionists need to consider what interventions can be taken in order to counteract the influence of non-professional and bad actors on social media.

## 1. Introduction

Over 2.8 million people die each year from being overweight or obese, a largely preventable disease [1]. Worldwide obestity has nearly tripled since 1975 [1]. In addition to loss of life, obesity places a substantial burden on society and health systems, and it contributes to lost economic productivity and reduced quality of life [2]. Public health education and the promotion of healthy diets combined with physical activity is a critical public health strategy in the mitigation of adverse effects that are associated with being overweight or obese.

Social media has fundamentally changed the way we communicate, collaborate, consume, and create content [3]. Unsurprisingly, it has emerged as a major source of health information [4,5,6]. The public both consume and share content, not only from the traditional more authoritative public health information sources, but also from their own experience, as well as personal and commercial sources. In addition, the ease with which content can be shared has resulted in a rapid increase in the number of individuals or organisations that seek to influence opinion, and the volume of content that they generate. These include influentials, or as more often called in the popular media influencers. Health information on social media is not subject to the same degree of filtering and quality control by professional gatekeepers common in either public health or commercial sources [7]. In addition, it is prone to being out of date, incomplete, and inaccurate [7]. The nutrition and diet domain is not immune to this phenomenon. In many cases, content may be shared and promoted without accepted scientific evidence or it is contrary to public policy and, in extreme cases, may be deceptive, unethical, and misleading [8,9,10].

The objective of this paper is to improve our understanding of Twitter in the healthy diet context, and to a limited extent, social media in general. In particular, we seek to (i) identify the most influential users in the healthy diet discourse on Twitter and explore the characteristics of these users, and (ii) identify most prevalent topics and sub-topics in the healthy diet discourse on Twitter. This paper presents the results of a retrospective exploratory data analysis of 1,212,318 million English language tweets featuring the phrase ‘healthy diet’ or the hashtag #healthydiet published over a 16-month period from January 2018 to April 2019. We use a novel multi-component method, including descriptive, content, and network analytics to identify and categorise users and content.

## 2. Background

### 2.1. The Rise of Social Media Influencers

There is a long and well-established literature on word of mouth (WOM) marketing, going back to the mid-nineteenth century. It encompasses opinion leaders [11], early adopters (including influentials and imitators) [12,13], market mavens [14], and hubs [15]. These categories all describe individuals who have above average ability to informally influence the attitudes and behaviours of others in a desired way [12]. They may vary in terms of their stage of adoption, product knowledge, or social connectedness, but they are categorised by their above average reach (i.e., the net number or percentage of people exposed, at least once, to a particular piece of content during a given period [16]) and impact [17,18]. At a high level, their influence is a combination of personal and social attributes—(i) the personification of certain values (“who one is”); (ii) competence (“what one knows”); and (iii) strategic social location (“whom one knows”) [11]. The combinatorial strength of these attributes determines a given influencer’s communicative power [19].

Historically, a given influencer’s communicative power was limited by their ability to reach their network or audience. Their role and impact was passive, offline, and, consequently, their impact limited. Web 2.0 provided both influencers and the wider general public with a platform to both consume and produce content with significantly greater reach. In addition, social media enables ordinary users to easily and quickly discover others with similar interests worldwide (e.g., through hashtags and search) and furnishes highly visible social signals of representing popularity (e.g., follower counts) and endorsement (e.g., likes, comments, and shares) [20]. In the absence of any pre-existing awareness or relationship, personal or otherwise, with another user, social media users make self-judgements on whether to trust and be influenced by other social media users based on these signals. User profile descriptions, content, and other more structured and quantitative data provide receivers with indications of the values, knowledge, and network of other users [21]. Similarly, prospective influencers can craft a personal brand for their account based on these signals [22]. Over time, such personal brands can become a form of micro-celebrity or micro-influencer through sustained social media interaction and engagement [23]. Research consistently suggests that electronic word of mouth (eWOM) can amplify reach and sales [24]. Influence marketing is a significant part of the marketing mix and it is widely used by brands worldwide, a full-time job for many, and an aspiration for many more. By 2017, over 75% of US advertisers employed some form of influencer marketing [25]; it is a billion dollar concern [26]. Unsurprisingly, given this revenue opportunity, brands and influencers make use of a wide array of professional and advanced techniques to present themselves and their content, engage with, and extend, their audience, and measure their impact.

In 1976, Campbell [27] famously stated: “the more any quantitative social indicator is used for social decision-making, the more subject it will be to corruption pressures and the more apt it will be to distort and corrupt the social processes it is intended to monitor”.

Attracted by fame and fortune, the intensity and level of competition in the influencer market has exploded. Unfortunately, so has the use of tools and techniques to manipulate social signals. Targeted advertising, automation (including spamming), low cost labour in developing countries, and black hat techniques, including followback networks, bought followers, bots, and other unethical, fraudulent, or deceptive practices, are features of the influencer market [28,29,30].

### 2.2. The Nutrition and Diet Discourse on Social Media

The general public use social media widely to consume and share nutrition and diet content. It is not only changing health seeking behaviours, but attitudes towards food and how it is purchased, prepared, and eaten. For example, research suggests that, for many people, social media plays a significant role in inspiring food choice, often through photoimagery [10], and food preparation (e.g., through “how to” videos and posts) [31]. Popular social media content on food, nutrition, and diet is often promoted by celebrities, dietitians, advocates of special diet, and weight loss programs, amongst others [32]. A wide range of influencers are active in this discourse. These include (i) everyday influencers (e.g., family and friends), (ii) micro, professional, macro, and celebrity influencers who found popularity on social media (e.g., @thecuttingveg), and (iii) celebrity influencers who found popularity on traditional media (e.g., Kim Kardashian) [32,33]. Eysenbach [34] posits that this change in health information seeking strategy reflects a shift from intermediation to apomediation. Whereas, intermediation that is involved traditional experts and authorities acting as gatekeepers between the public and information, apomediation is a health information seeking strategy where the public receive “guidance” from the crowd, peers, and others, using networked collaborative filtering processes, and without the same restrictions in access to information [34].

The democratisation of medicine and health using Web 2.0 technologies is heralded widely. Notwithstanding this, research suggests that there are significant concerns regarding information inaccuracy and potential risks that are associated with the use of inaccurate health information, amongst others [35,36,37]. While commercial and public health sources are constrained by regulations, social media health information shared online is prone to being out of date, incomplete and inaccurate [7]. Social media posts relating to nutrition and diet may promote pharmaceutical treatments for weight loss lacking detail on potential side-effects when a drug should be prescribed [38], or promote a dietary pattern without evidence of long-term safety and efficacy within the general population [32]. Two recent Australian cases involving social media influencers, Jess Ainscough and Belle Gibson, are instructive. Both built significant profiles as wellness influencers while advocating controversial and outdated therapies, including the Gerson therapy, to cure cancer; Ainscough succumbed to her illness, while it appears that Gibson never had cancer in the first place [20]. Recent research, albeit on a small sample of social media influencers, found that the influencers studied were inadequately qualified, presented opinion as fact, and were contrary to nutritional guidelines [39]. The consequences of promoting faddism, misinformation, and misinterpretations is well documented and include delay or failure to seek or continue legitimate medical treatment, malnutrition, and interference with sound nutrition education and public health policy [8,40].

### 2.3. Computational Analysis of the Nutrition and Diet Discourse on Twitter

Discussions of Medicine 2.0 and Health 2.0 feature a number of common themes, which include the use of new Web 2.0 technologies characterised by social networking, participation, apomediation, openness, and collaboration [34,36]. These attributes combined with the sheer volume of active social media users enables new forms of health surveillance and research [41,42]. Twitter, despite not being representative of the general population [43], is increasingly used for health surveillance and research [44]. Firstly, it is actively used by a substantial number of users from a wide variety of contexts. During the period under examination in this study (2018–2019), the median monthly monetisable daily active usage on Twitter was 125 million users worldwide [45]. US data suggest that Twitter users are active on multiple other social networks and reflect a wide range of demographics [38]. Secondly, unlike other popular social networking sites, Twitter is largely an open network and, as such, facilitates the connection, sharing, and consumption of content between both acquaintances and strangers [21]. Thirdly, hashtags (#), widely used on Twitter, play a key role in enabling Twitter users to identify content and other users with similar and opposing views, and form ad hoc publics and communities around a specific hashtag [46]. Fourthly, Twitter data not only comprise the core data, e.g., the user and tweet content, but so-called data exhaust, ambient data passively collected by Twitter in the operation of the platform. For example, this includes data on the hardware and software that a given Twitter account used to generate a given tweet.

Extant research on the nutrition and diet discourse on social media typically focuses on small datasets that are manually coded. By its nature, Twitter generates Big Data; data whose volume, variety, and velocity are at orders of magnitude greater than traditional health surveillance and research methods. These characteristics, the mix of structured and unstructured data, and high dimensionality require new computational methods for analysis, such as machine learning. These techniques are used in order to identify patterns and relationships in large data sets. They are increasingly used in nutrition and diet research including geo-spatial analysis [47], temporal analysis [48], user identification [46,49], content analysis [46,48,49,50], and network (hub) analysis [49].

Widener and Wenwen [47] use a dataset of of 128,914 tweets from one month. While using mixture of classification and sentiment analysis, they explored how geolocated tweets could be used to map the prevalence of of healthy and unhealthy food across the US. He and Luo [46] used an associative classification algorithm to identify pro-eating disorder posts and users on Tumblr and Twitter. As well as achieving high accuracy, they found that such posts featured co-occurring hashtags, such as #thinspiration, #weightloss, #skinny, and other beauty and body image hashtags. Turner-McGrievy and Beets [48] examine temporal trends in Twitter posts about weight loss by tracking mentions of the four hashtags (#weightloss, #fitness, #diet, and #health) with “weight” in the posts in different pre- and post holiday time frames over a 1-year period. They find that (i) people are discussing weight loss during and after holidays and during the winter when weight gain commonly occurs, and (ii) the discourse changes over a year and is impacted by seasonal trends.

Eriksson-Backa et al. [49] analysed 607,905 tweets containing the word ‘diet’ for one month and then analysed only those tweets related to diabetes. They found a wide range users types including media, commercial and public health organisations. Network analysis was used in order to identify hubs; they found that public health organisations had the highest in-degree (mentions by others), while the highest out-degrees were accounts for news dissemination and public health organisations. They provided confirmatory evidence that the public were active participants in the diabetes discourse and acted as disseminators of information and news about diabetes and diets. Karami et al. [50] analysed a dataset of 4.5 million tweets for one month to discover and analyse topics on Diabetes, Diet, Exercise, and Obesity (DDEO). They found strong correlations between the DDEO topics. Within topics, users discussed a wide range of topics including health conditions (e.g., pregnancy and mental health), celebrities, weight loss, and religion. More recently, Yeruva et al. [51] used a pipeline model in order to explore the relationship between obesity and healthy eating to compare the the Twitter discourse with expert discourse, sourced from PubMed. Using a variety of machine learning techniques including Term Frequency and Inverse Document Frequency (TF-IDF), Word2Vec, Natural Language Processing (NLP), sentiment analysis, and Latent Dirichlet Allocation (LDA), amongst others, they find a significant variance between both groups in terms of the topics discussed and each cohorts’ perspectives of healthy eating and obesity.

### 2.4. Research Questions

While studies using large datasets are emerging, these are typically limited by restrictions from using the free Twitter API or third party aggregators. They are often constrained to small time frames, one month, and, as such, their findings may not be suited for wider generalisation, as they do not allow for seasonality or changes in public opinion [48]. Typically, the focus is on one particular analytical technique, often content analysis, without associated analysis of the actors, the role and influence of different actors in the discourse, and the provenance of the content publisher or the quality of the content itself.

In this study, we focus on the healthy diet discourse on Twitter. Extant research suggests that the general public are familiar with the general principles of a healthy eating and related concepts but define or interpret this in different ways, depending on their individual context and demographics [52]. Similarly, the concept of a healthy diet has evolved over time and is highly inflected by the historical and cultural context in which they are produced, the entity promoting a given healthy diet message, and the individual beliefs, motivations and context of those receiving the message [53,54]. As such, a precise definition of a healthy diet can seem vague and abstract, and, consequently, may lead to confusion [52,54]. Ristovski-Slijepcevic et al. [52] suggest that notions of healthy eating can be seen as representations that are conveyed through discourses. These may be official, commercial, or personal discourses or a combination of all three.

In effect there are two categories of accounts on Twitter—humans and automated software programs or ‘bots’. These categories are not binary, a third category, cyborgs, also exists and includes bot-assisted humans and human-assisted social bots [55]. Research on social bots (hereafter also refereed to as “bots”) suggest that they retweet more than humans, have longer user names, and generate fewer replies, retweets, and mentions from human Twitter users [56]. In 2017, Varol et al. [57] estimated that between 9% and 15% of active Twitter accounts were bots. Social bots may be instrumental or communicative [21]. For example, instrumental social bots include automated posts from activity trackers that are connected to user Twitter accounts, such as FitBit, or the use of enterprise marketing technologies, such as Hootsuite. In contrast, communicative social bots seek to mimic human communications and, while using machine learning, may be interactive to some degree [58]. Obviously, the use of social bots can be benign. For example, social bots are used for health activity tracking, marketing productivity, customer service, or news feeds [59]. Unfortunately, they can also be malicious; social bots are widely used for spamming, manipulative marketing, impersonation, and distributing malware [59]. More recently, the media and research has highlighted the use of social bots to influence Twitter discourses. Such bots may be used individually or in social botnets, a form of ‘sock puppetry’ [60]. Social botnets are extremely difficult to identify, as the network comprises real users. They typically include large groups of bots under the control of a single coordinator (botmaster) who coordinates their interactions. These can be used to generate spam tweets independently of each other or as a single retweeting tree or retweet chain [59]. We know that, in other contexts, particularly in political discourse, there is increasing and widespread evidence of bots to influence opinion or distract the general public through a variety of practices, including (i) astroturfing, a form of manufactured top-down activity on the Internet that is designed to mimic bottom-up activity by autonomous individuals with the intent to deceive the public at large that the activity is real [61]; (ii) smoke screening, where a bot network uses a high volume of content, replete with related hashtags and keywords, to de-emphasise or obscure some other type of activity or content [62]; and (iii) misdirection, where a bot network uses a high volume of content, to get the public to focus elsewhere [59,62]. Ultimately, these practices all serve to confuse the general public by presenting a particular viewpoint as being more popular or widely accepted than it is in reality by spreading misinformation, and/or distract them from authentic evidence-based messages by generating noise. Although there is limited research on bots in health contexts, studies identified their use in promoting anti-vaccination [63] and e-cigarettes [64]. In their call for research on curbing the spread of health misinformation, Pagoto et al. [37] specifically call for research on the identification of false messaging, messengers and their motivations, and the mechanisms that they use to generate and disseminate false messages. In this paper, we present an early, if not first, attempt to address these questions in the nutrition and diet domain.

This paper attempts to make sense of how different actors engage in the healthy diet discourse on one social media platform, Twitter, over a 16-month period. It has two objectives. First, we seek to understand the characteristics of the most influential users in the healthy diet discourse. Secondly, we wish to identify and explore the most prevalent topics and sub-topics discussed in the healthy diet Twitter discourse over a prolonged period. In line with [21,65], we perform an exploratory data analysis while using descriptive, content, and network analytics to answer the following research questions:

1. Who are the most influential users in the healthy diet discourse on Twitter? What are the characteristics of these users? Is there evidence of attempts to manipulate or deceive the general public?
2. What are the most prevalent topics and sub-topics in the healthy diet discourse over a 16 month period?

## 3. Data and Methods

### 3.1. Data Collection

Using Twitter’s enterprise API platform, GNIP, we prepared a dataset of all English language tweets featuring the phrase ‘healthy diet’ and hashtag ‘#healthydiet’ from 1 January 2018 to 30 April 2019. The duration of the data collection was motivated by three discrete factors (i) collecting data for an entire calendar year, (ii) having data from a second period on which to test the generalisability of models built on the first period, and (iii) budgetary constraints. Table 1 provides an overview of the dataset used in this study. This includes 1,212,318 tweets posted by 629,608 discrete Twitter user screen-names or user accounts. 45% of the tweets are original posts, 48% are retweets, while the remaining 7% are replies. 7300 out of the 629,608 (1.2%) of the users in our dataset are verified.

**Figure 1:**
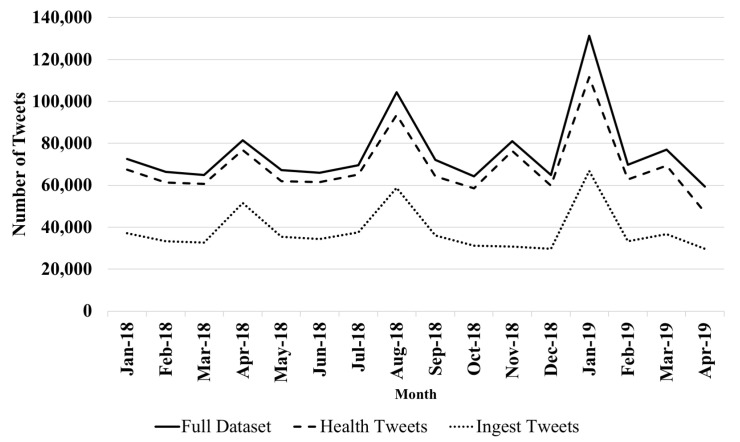
Monthly Volume of Tweets.

Table 2 provides the list of the top 20 countries (country information is only available for 59% of the tweets) in terms of volume of tweets. The largest volume of tweets were posted from US and UK users that account for 37% of the total volume of tweets. Figure 1 visualises the monthly volume of tweets during the 16 months that were covered by our dataset for the full dataset and for tweets associated with Health and Ingest topics. As expected, the graph suggests that people discuss nutrition and diet throughout the year and exhibit changes throughout the year, particularly during the seasonal holiday periods and in June/July and December/January. The trend line in the graph also suggests a growing interest in the healthy diet discussion over time. These findings are consistent with extant research on weight loss discussions on Twitter [48].

### 3.2. Methods

In this study, we implement exploratory data analysis [66], an approach that has been widely used in social media research, at it allows data to “speak for itself” and enables researchers to identify and navigate particular aspects of interest [67,68,69,70]. By combining statistics, machine learning, and data visualisation techniques, exploratory data analysis goes beyond formal modeling or hypothesis testing and lets patterns, trends, and relations in data emerge [71].

#### 3.2.1. User Analysis

We performed two discrete analyses in order to assess the prevalence of professional and manipulative messaging techniques. First, to explore the sophistication of technologies used in the discourse, we examined the type of software used to generate tweets. We use the generator metadata available from GNIP to identify the software utility that was used to post the Tweet. This metadata includes the name and a link for the source application that generated the tweet. The general public typically use official Twitter clients or other social networking platforms for cross-posting (e.g., Instagram, Facebook, etc.), while commercial actors are more likely to use marketing automation software. The generator metadata can provide evidence of bot applications. The IUNI Botometer (formerly BotOrNot) was used to identify social bots. Botometer is a machine learning algorithm for detecting social bots on Twitter; it has a reported social bot detection accuracy in excess of 95% [56,72]. To detect potential social bots, Botometer leverages 1000 features from a Twitter account and its activity in order to evaluate the similarity of that account to the known features of social bots [57]. These include user-based, friends, network, temporal, content and language, and sentiment features, amongst others [57].

In order to identify influential users, we implemented three complementary approaches. First, we identified verified accounts. Verified accounts are accounts that have been authenticated or has been determined to be an account of public interest by Twitter. Such accounts display a blue verified badge next to the name on an account’s profile and next to the account name in search results. We identified these accounts while using the verified metadata sourced from GNIP. Second, we explored users’ activity and visibility as this may indicate influence [73]. Activity is measured as the sum of tweets, retweets, and replies posted by a user while visibility is measured as the number of retweets and replies received by a user [65]. Third, we used network analytics techniques in order to capture the relational dynamics between the users in the healthy diet network and to identify the most influential users. Specifically, we (i) constructed a network based on reply links, as these are typically associated with the start of a conversation [74] and, therefore, represent stronger connections than retweets [75]; (ii) used the Force Atlas 2 algorithm to define the layout of the network [76]; (iii) implemented the Louvain method for community detection to identify sub-communities within the network [77]; and (iv) used the PageRank algorithm to identify influencers [78,79].

#### 3.2.2. Topic Content Analysis

The topic content analysis was conducted while using a lexicon-based approach leveraging pre-compiled dictionaries to group words in a topic [50]. For the purpose of this study, we used the Health and Ingest dictionaries available in the Linguistic Inquiry and Word Count (LIWC) 2015 [80]. LIWC is widely used in academic research and its validity and reliability have been tested in multiple domains [81]. Hashtags and URLs were first removed from the corpus of tweets, and then a lexicon based classifier was used in order to count the frequency of occurrence of each word listed in the three dictionaries mentioned above. The topic content analysis was only performed on original tweets, as the focus is on what users tweeted about rather than the extent that they were amplified, or whether the account engaged in subsequent conversations.

## 4. Results

### 4.1. Who Are the Most Influential Users in the Healthy Diet Discourse on Twitter?

#### 4.1.1. Of Verified, Bots, and Suspended Accounts

Our first research question sought to extend and deepen our current understanding of participants in the healthy diet discourse on Twitter and the characteristics of these users. Social influence is a combination of who the social actor is, what they know about the focal topic, and who they know, as discussed in Section 2. Their communicative power is highly influenced by their direct reach (i.e., the followers of that account) and indirect reach, all those who do not follow but can access a message through retweets, hashtags, search, or other clients through which Twitter is syndicated (the Twitter fabric). With regards to who the social actors is—there are four account categories worthy of immediate exploration—verified accounts, bots, suspended accounts, and accounts that are not verified, bots or suspended. Research suggests that verified status increases both perceived trustworthiness and general source credibility [82,83]. In contrast, bots may be accounts operated by humans but augmented by software, or automated accounts for instrumental or communicative purposes, some of which are designed to mimic humans, and only exist to amplify reach or manipulate target users. Suspended accounts are accounts that Twitter has deemed to be unsafe because they break Twitter Rules. These include spam, fake, or abusive accounts, or accounts that impersonate other accounts.

Our study included 629,608 discrete Twitter accounts, 7300 (1.1%) of which were verified users. These include government and public institutions (e.g., WHO), politicians (e.g., Donald Trump), celebrities (e.g., Kim Kardashian), general and special interest media (e.g., The Huffington Post and Men’s Health Magazine), and other high profile accounts in key interest areas (e.g., the South Beach Diet). While verified status tells us who they are, it should be noted that this does not necessarily make them any more competent or credible on health, nutrition, and diet, or consistent with public policy.

With regards to bots and suspended accounts, we analysed the top one hundred active and visible accounts in the dataset while using the Botometer algorithm. The results suggest that 81% or more presented high similarities to bots (60%) or had been suspended by Twitter (21%). Of the remainder, only 7% of the top 100 active accounts had a low or very low similarity to bots. In contrast, highly visible users tended to have low similarities to bots (86%); of the remaining 14%, eight accounts were suspended. Table 3 summarises these findings.

Our analysis of the generator metadata confirms both our expectations in that (i) the main client used by participants in the healthy diet discourse are official Twitter clients (e.g., Twitter Web, Twitter Lite, iPhone, Android, iPad, WebApp, TweetDeck, etc.), and (ii) there is widespread use of enterprise marketing automation software (e.g., Hootsuite, Edge Theory, Social Oomph), and bot generators (e.g., IFTTT and Bot Libre!). Indeed there is an significant long tail of other generators used in the healthy diet discourse, including over 400 bot generators. Table 4 summarises the top ten generators in the healthy diet data set.

The impact of low quality accounts in creating noise and confusion in the healthy diet discourse should not be underestimated. For example, over 103,626 tweets in the data set were generated by (i) accounts in the top 100 most active users categorised as being highly similar to bots or suspended (88,860 tweets), or (ii) other accounts not in (i) that use generators featuring “bot” in their display name (14,766 tweets). This suggests at least 8.5% of the discourse is generated by computers as social actors. This is a conservative estimate, as the Botometer classifier is not completely accurate and some of the generators may be bots even if their name does not say it (e.g., IFTTT). This is consistent with previously cited studies suggesting that bots make up 9% to 15% of tweets [57]. Further analysis identifies nearly 151,183 (28%) original tweets that feature spam characteristics. These include blatant digital astroturfing and, to a lesser extent, misdirection. For example, one highly active account featuring hashtags related to dieting and links to a gateway site featuring links to health insurance, medical billing services, electronic medical records, and weight loss programmes, amongst others. This leads to a page featuring multiple Google Ads advertisements, from many high profile rival health brands. Another highly active account linked to an inactive click farm.

Once we remove bots, suspended accounts, and accounts generating spam, we find a population that is more representative of the general Twitter population comprising the general public, celebrities, commercial accounts, public service institutions, subject matter experts, etc.

#### 4.1.2. Active vs. Visible Accounts

Early studies regarding influence on Twitter suggested that the most active and the most visible users may indicate influence on Twitter [73]. Subsequent studies, in both commercial and non-commercial contexts, highlight qualitative differences in the legitimacy of highly active users when compared to highly visible users [82,84,85,86,87]. By and large, these studies suggest that highly active accounts are more likely to be characterised as automated and they are less likely and unlikely to be the most visible users, and vice-versa. For the purpose of this study, the most active users are those accounts who generate the greatest volume of tweets, retweets, and replies, while the most visible users are those accounts with the greatest volume of retweets and replies.

**Figure 2:**
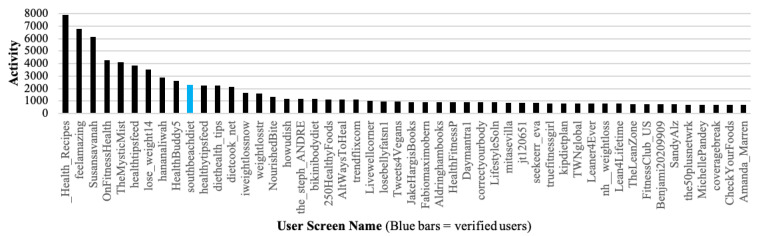
Most Active Users.

**Figure 3:**
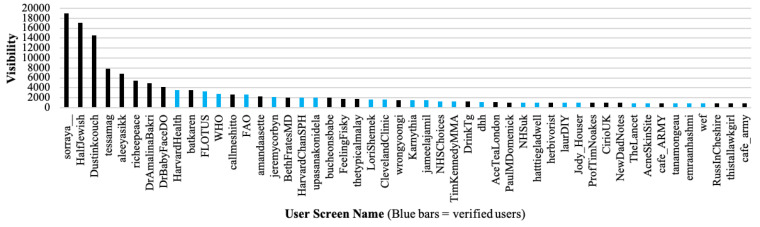
Most Visible Users.

Figure 2 charts the 50 most active users and their associated level of activity. There is clearly a small group of users who are extremely active and a long tail of users who posted less than 1000 tweets (less than two tweets per day on average) during the 16 months covered by our data set. The graph also shows that only one out of the 50 most active users is verified (blue bar). Figure 3 charts the 50 most visible users and their corresponding level of visibility. The results highlight the fact that there is a relatively small number of highly visible users who attract significant attention and a large number of users who are not as influential. Analysis of highly active and visible accounts for verified status, bots, and suspended accounts provides further confirmatory evidence regarding the legitimacy of highly visible accounts vs highly active accounts. Firstly, there is very little overlap between highly active and highly visible accounts in the healthy diet data set. Secondly, the 50 most visible accounts feature a significantly greater proportion of verified status accounts as compared to the 50 most active accounts suggesting that the former are more credible than the latter. Thirdly, the top 50 most active accounts are more likely to use enterprise marketing or automated tools, have similar characteristics to bots, feature, or are connected to, spam accounts, and feature a higher proportion of post facto suspended accounts.

### 4.2. Network Analysis

**Figure 4:**
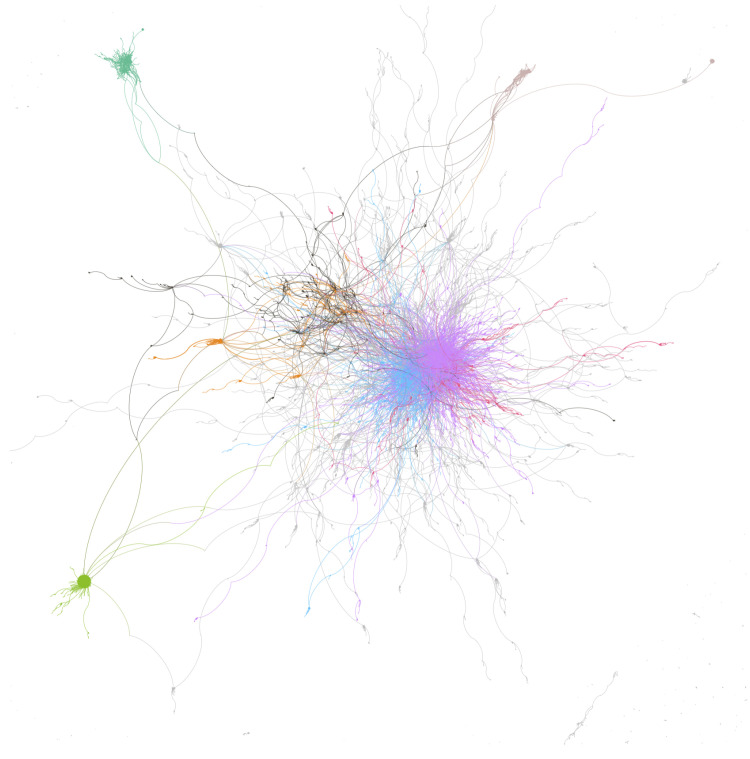
*Healthy Diet* Network Visualisation.

Katz posited that the strategic social location of a focal person contributes to influence [11]. As such, influential users may also be inferred while using network analytics. These techniques explore how users interact within an online community and how they cluster together in sub-communities. Social networks are made of nodes (i.e., users) and edges (i.e., links) between users. The healthy diet network on Twitter has 114,190 nodes (users) and 77,725 edges (replies) (see Figure 4). The network has a diameter of 31. This means that the two most distant nodes in the network are 31 users apart from each other, therefore suggesting that the network is highly sparse. This is also reflected in the average path length (i.e., the average distance between nodes), which is equal to 7.94. A short average path length would suggest the presence of highly influential users in the network. Here, the distance is relatively high, nearly eight, suggesting relatively few network brokers or hubs. The average degree (the average number of connections per node) is 1.36; each node in this network has received only 1.36 replies from within the network. This provides supporting evidence that the healthy diet data set has a small number of popular brokers and hubs. As a whole, these results indicate that the overall structure of the healthy diet network does not really facilitate connections in a substantive way, nor is it exploited effectively.

Chae [65] suggested that the degree of connectedness of a given node in a Twitter network can be an indicator of a user’s popularity and, consequently, their influence. Influential users are typically characterised by a large number of incoming connections (in-degree) and a low number of outgoing connections (out-degree). In online social networks such as Twitter, these measures can be identified using their PageRank score i.e., the ratio between incoming and outgoing connections as well as the influence of these connections [78,79]. Table 5 presents the results for the 10 most influential users in the network. These include commercial and non-commercial promoters of special diets and related services, fitness services, and pharmacy products, a politician, and a UK television programme. Interestingly, two of the most influential accounts, the South Beach Diet and PETA, while differing in motivation, promote specific special diet regimes, the former a branded low carbohydrate diet, and the latter, a vegan diet and lifestyle. Of the ten influencers, only one, Susan Hart Nutrition (@SH_Nutrition), is an independent qualified nutritional coach. Notwithstanding that, @SH_Nutrition, while stating that she is a nutrition coach does not provide any indication of her qualifications in her profile; this is only apparent with additional research.

**Figure 5:**
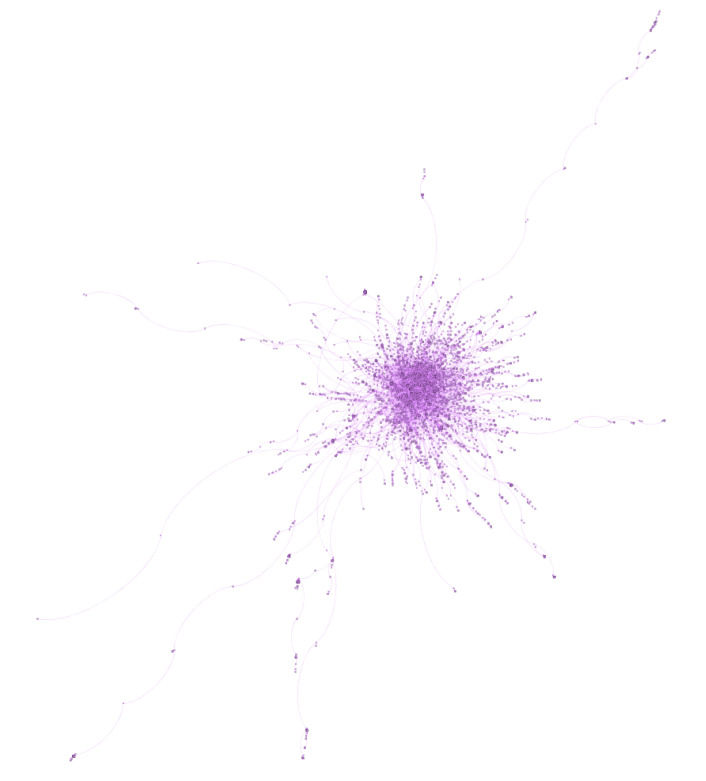
SC1 is the largest sub-community and is a more general and distributed community.

As discussed, an analysis of the healthy diet network as a whole did not present evidence of strong influencers or brokers. The modularity score of the network (0.974) though suggests that interactions within the sub-communities in the network are much stronger than the interactions between sub-communities and, therefore, at a network level. With this in mind, we identified the five largest sub-communities within the healthy diet data set. The largest sub-community (SC1) comprises 4740 users (Figure 5) and appears to be built around information sharing about different special diets (e.g., low carbohydrate high protein, vegan/vegetarian etc.), and healthy lifestyle more generally. No single account dominates SC1, rather influence is distributed across a number of accounts with heterogeneous backgrounds reflecting a more authentic community. While the profiles of top 10 most prominent users based on network analysis include a film producer (@AmandaZZ100), two academics (@ProfTimNoakes and @drjkahn), an athlete (@SBakerMD), a science journalist and author (@bigfatsurprise), and bloggers (e.g., @theveganparent, @Mangan150, @MacroFour); each of these accounts typically promotes some form special dietary pattern. For example, @ProfTimNoakes, @SBakerMD, @bigfatsurprise, @AmandaZZ100, @Mangan150, and @MacroFour are all active promoters of low carbohydate, high fat, and/or high protein dietary patterns, while @drjkahn and @theveganparent actively promote vegan and vegetarian dietary patterns.

**Figure 6:**
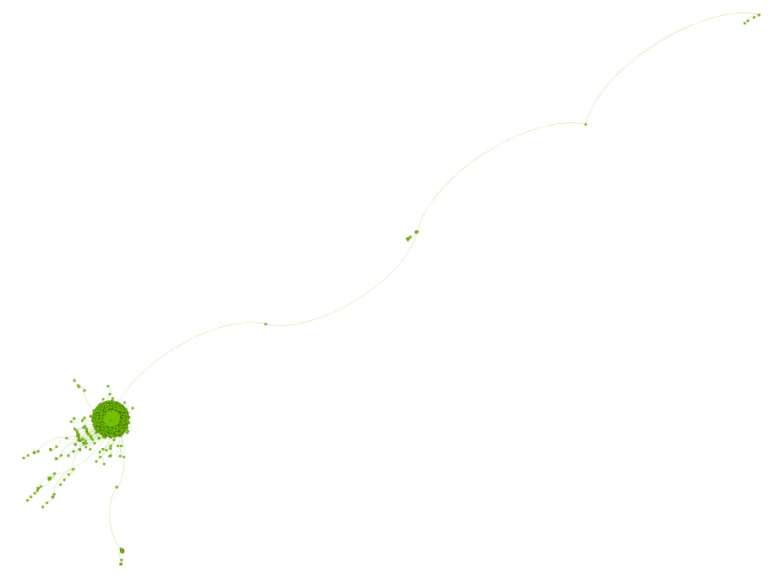
SC2 is the second largest sub-community and is centred on the South Beach Diet.

**Figure 7:**
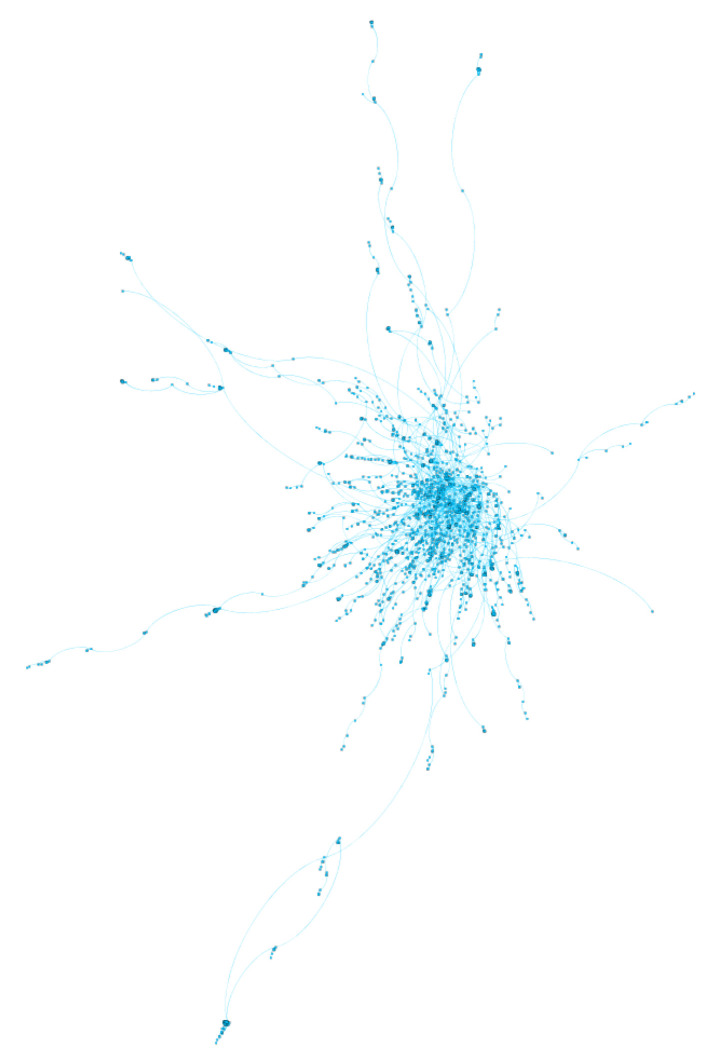
SC3 is the third largest sub-community is centred on vegan diet and lifestyle.

The second largest sub-community (SC2—Figure 6) is half the size of SC1 with 2347 users and is clearly structured around one key influencer (@southbeachdiet), which accounts for 96% of the connections within the community. Consequently, the vast majority of the discourse focus on the products and ketogenic recipes related to this diet. Similarly, the third largest sub-community (SC3—Figure 7) with 1936 users is organised around vegan diet and lifestyle. Of the top 10 most influential users in SC3, only one claims to be a qualified nutritionist (@vegannutrition1). SC2 and SC3 are consistent and they provide explanatory value with respect to the network-level findings discussed earlier.

**Figure 8:**
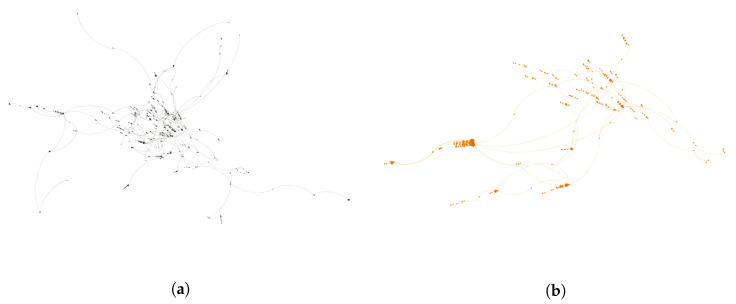
(**a**) SC4 is the fourth largest sub-community and is media-centred. (**b**) SC5 is the fifth largest sub-community and is centred on established public health organisations and qualified individuals. SC4 and SC5 are the fourth and fifth largest sub-communities in the *Healthy Diet* network.

The fourth largest sub-community (SC4—Figure 8a) and the fifth largest sub-community (SC5—Figure 8b) are much smaller in size when compared to SC1-SC3 with 987 and 823 users, respectively. However, in these sub-communities, the presence of conventional sources of health information and influencers can be identified. For example, the main influencers in SC4 are media-centred, including health specific publishers and media (e.g., Harvard Health Publishing, Men’s Health Magazine), mainstream traditional and new media (e.g., The Wall Street Journal and The Huffington Post), and celebrities (e.g., Kim and Khloe Kardashian). Similarly SC5, is centred around public health organisations such as the WHO, and the World Economic Forum. SC5 also features qualified individuals with either commercial profiles (e.g., Cristina Dragani, CEO of Eneksia, an Italian supplement company) or media profiles (e.g., Dr. Lori Shemek, a best-selling publisher and US media commentator). These SC5 examples illustrate the tension between public health agencies, such as the the US Office of Disease Prevention and Health Promotion and the UK NHS, and individual influencers. Typically, government policy recommends a healthy, balanced diet as the source of vitamins and minerals. Many public health authorities also recommend vitamin supplements for specific population cohorts e.g., folic acid for women trying to conceive or in early stages of pregnancy, vitamin D for older people, etc. On the other hand, few public health authorities recommend supplements for weight-loss and, indeed, a review of the research suggests that many of the health claims for supplements are unfounded or lack substantive evidence of benefits [88]. Similarly, public health authorities and nutritionist bodies, while outlining the benefits of special diets in specific circumstances, also highlight the limitations, challenges, and adverse effects of both special diet and weight-loss supplements [88,89,90].

### 4.3. What Are the Most Prevalent Topics and Sub-Topics in the Healthy Diet Discourse?

Table 6, Table 7, Table 8 and Table 9 present the frequency of different topics that are mentioned in original messages, the subset of spam tweets (i.e. tweets featuring very similar text posted repeatedly by the same user), original messages with no spam, and original messages posted by verified users, respectively. Topics were isolated using a lexicon-based approached based the Linguistic Inquiry and Word Count (LIWC) 2015 dictionaries for Health and Ingest.

Unsurprisingly, given the focus of the data set, diet is by far the most discussed sub-topic in both Health and Ingest topics across all account categories, accounting for typically 8–10 times the tweet volume in Health and 3–4 times the tweet volume in Ingest. In Health, common sub-topics in all categories include nutrition and disease-related tweets, specifically diabetes. In verified and general account categories, cancer is also relatively prominent as a sub-topic. Interestingly, fat(s) does not feature in the top ten sub-topics by verified users but does in the general population, and significantly so in the spam account category. Indeed, the topics that are discussed in Health for verified and general accounts are more similar than spam accounts. Spam accounts emphasise fat(s) more, but also very specific emotive appeals and topics e.g., healing and pregnancy sub-topics. It is noteworthy, that obesity is not a prominent topic in any account category. This is consistent with previous research [51].

Ingest sub-topics are quite similar across all account categories, with diet, food, eat/eating, and weight being the most prominent sub-topics in all categories, although weight is a more prominent sub-topic in tweets generated by spam accounts. Tweets about fat(s) are again less prominent in verified accounts; discussion on snacks are less prominent in general accounts.

We sought to classify tweets based on the prominence of words that are related to well-known special diets, including high protein/low or no carbohydrate, vegan/vegetarian, gluten free, dairy free, high carbohydrate, and other dietary regimes not classified elsewhere, given the prevalence of diet as a sub-topic in the data set and the prevalence of dieting as a common behaviour in society (Table 10). Firstly, it is surprising how few tweets reference the language of popular diets specifically in this data set. This may be due to the focus of the discourse or, as is more likely, more relatable language is used. Secondly, notwithstanding the low volume of tweets, two significant clusters emerge high protein/low or no carbohydrate diets and vegan/vegetarian diets. For the former, ketogenic diets are significantly more prominent; this is consistent with our network analysis that suggests and are findings related to the influence of the South Beach Diet, a popular ketogenic diets. These diets are also more prominent in tweets by spam accounts, most likely due to the prevalence of affiliate marketing programmes. In contrast, vegan/vegetarian diet are marginally more prominent in tweets by verified accounts, although the overall number of tweets is small in number.

## 5. Discussion

This objective of this research was to extend our understanding of the dynamics of the healthy diet discourse on Twitter by identifying the characteristics of the most influential actors and the most prevalent topics discussed. All too often, discussion on social media influencers is reduced to quantitative measures—the number of followers, the number of posts, the number of likes, and so on. In the absence of personal knowledge, one can understand how the public might rely on such signals. Notwithstanding this, the trustworthiness and credibility of these information sources should play a role and, where one’s health and well-being is at stake, it is not irrational to suggest that this role should be significant. In psychology, organisational trust is often presented as a multi-faceted construct comprising competence, integrity, and benevolence [91]. In this respect, trustworthiness and influence are somewhat similar. Those consuming, and potentially acting on, nutrition and diet content on social media open themselves up to vulnerability on the assumption that the information provider is competent, honest, and acting in their best interest. The quantitative signals and network linkages serve to reinforce this trustworthiness and reduce anxiety. Unfortunately, there evidence to suggest that a significant proportion of the content and accounts in the healthy diet should not be trusted at face value.

To a large extent, our analysis suggests that there are two dominant categories of actors in the healthy diet discourse—those disseminating a message, both publishing and amplifying, and those consuming that message. Over 80% of accounts fall in to the latter. When one takes in to account the user and network analysis in Section 4, one might posit that the healthy diet discourse is not driven top down by the influencers, but, as Watts and Dodds [92] suggest, by a critical mass of easily influenced individuals. Reaching these individuals is made difficult by the noise and techniques that are used by commercial entities and other interested parties to market their products and services (e.g., special diets) and promote their worldview (e.g., veganism), but also by entities engaged in unethical, and sometimes malicious, practices, including click fraud schemes, malware distribution, and misinformation and disinformation dissemination. We find extensive use of social bots and automated software in the healthy diet discourse. At worst, such messaging may influence people’s behaviours with adverse health consequences, at best, it serves to confuse and negate public health efforts. These may even include content polluters under the direction of commercial entities, lobbyists, and political operatives [63]. In line with Pagoto et al. [37], policymakers and public health communicators need to consider strategies in order to (i) curb the spread of false or inaccurate messaging and (ii) inoculate the public from this messaging. Such countermeasures may include improved public health communications, digital health, nutrition and food literacy, and platform monitoring and regulation.

This study specifically explores the discourse on special diets. Extant research suggests that such diets may be linked with higher rates of eating disorders, even when following such a diet for therapeutic reasons [93,94]. While research suggests that many of these diets have benefits in specific contexts, they are not without controversy, both from medical and public health communication perspectives. For example, D’Souza et al. [95] suggest that current evidence indicates that the ketogenic diet results in short-term weight loss and improvements in glucose metabolism, but highlights concerns regarding its dyslipidemic potential in the context of cardiovascular disease treatment. Similarly, Johansson et al. [96] found that fat intake and cholesterol levels in Northern Sweden increased significantly over the same time period that the promotion of low-carbohydrate high-fat diets increased. The latter study lead to a long standing conflict between promoters of low-carbohydrate high-fat diets and the Swedish National Food Agency [97,98]. Our findings suggest that the tension between public health policy and the promoters of special diets is exacerbated by social media, where the latter do not operate under the same editorial restrictions as the former, and may have access to greater marketing resources than public sector agencies.

### 5.1. Public Health Communications

Our research provides further confirmatory support and insights on the use of social networking sites as information exchanges that are related to health, and nutrition and diet specifically. Consistent with [32,33], the healthy diet discourse features a wide range of actors seeking to influence the general public, including passionate citizen advocates, influencers of all sizes (micro to macro), professional and otherwise, as well as traditional influencers, such as politicians, celebrities, etc. Unfortunately, the discourse is also heavily influenced by commercial and special interests, as well as malicious actors, who may or may not disclose their interests and linkages. In particular, communication in this public discourse on Twitter involves the use of sophisticated automated marketing and widespread use of bots. Such practices can serve to distract and confuse the public, as well as reduce the impact of legitimate public health communications. Health research on Twitter, including nutrition and diet research, tends to focus on content and not the actors involved. Where such research is undertaken, computers as social actors are often neglected, often to the detriment of the research, and ultimately society [63].

Ultimately, public health communications are trying to persuade the public to adopt healthier behaviours and lifestyles. Cialdini [99] suggests that there are six principles of persuasion:

1. Reciprocity—people are more willing to comply with requests from those who have provided such things first.
2. Authority—people are more willing to follow the directions or recommendations of a communicator to whom they attribute relevant expertise.
3. Social Proof—people are more willing to take a recommended action if they see evidence that many others, especially similar others, are taking it.
4. Commitment and Consistency—people are more willing to be moved in a particular direction if they see it as consistent with an existing commitment (or world view).
5. Liking—people are more likely to comply with requests to those they know and like.
6. Scarcity—people find objects and opportunities more attractive to the degree that they are scarce rare, or dwindling in availability.

Many of the enterprise marketers, influencers, and content polluters in the healthy diet discourse optimise their social media presence around activities that highlight these factors. While public health organisations are authoritative and consistent, they do not act like humans and relatively speaking are not as engaging, visible, likeable, and, as a result, do not have the same social proofs with target audiences. The study of social media actors, their communications and messaging strategies, and the mechanisms that they use to leverage the functionality of social media using automated means, can help to develop more effective communications, counter-messaging strategies, and policy interventions. At a practical level, these might include:

- Segment—it may be difficult for the general public to relate to a large monolithic brand such as the WHO, WEF, and the NHS, whose operations are so wide that the general public either do not associate them with nutrition and diet or the content feed is not targeted enough for individual users. Such organisations need to consider whether it is more prudent to develop segment-specific accounts that are focused on nutrition or even sub-topics, where they can build and interact with a specific audience more specifically.
- Humanise—public health organisations and experts need to humanize their messaging and engagement so that it is a dialogue and not merely a public service announcement. This includes identifying individual users, developing a rapport, and maintaining contact, while at the same time presenting evidence-backed information and advice.
- Adapt—one of the challenges in social media is the network and other resources that influential accounts and botmasters control and have access to. Targeting influential accounts with high centrality to a community and discourse is unlikely to be successful and may result in backfire effects and give the target more prominence [100]. Our analysis suggests that the vast majority of participants in the healthy diet discourse did not have strong connections with others, these people are likely to be more receptive than highly active participants. Public health communicators need to fully use the arsenal of tactics at their command including non-confrontational skeptical questioning, providing alternative narratives and social proofs, and framing healthier alternatives or information in a positive way that is congruent with the target audience worldview [100].
- Belong—our research identified specific sub-communities in the healthy diet discourse organised around specific accounts and sub-topics. Public health sector organisations need to be authentic members of these communities through participation. Many members may be skeptical of such participation due to a variety of reasons including social reactance, existing belief systems and worldviews, literacy, sunk investment (psychological, physical and financial), and negative consequences of changing their position [37,100]. As such, trust needs to be built up over time through demonstrating consistent commitment to participate in the community.
- Attract—research suggests that brand personality content is associated with higher levels of social media engagement with a message, while directly informative content is associated with lower levels of engagement [101]. Pilgrim and Bohnet-Joschko [10] suggest that some of the success of nutrition and diet influencers can be partly explained by their communicative process, built on carefully designed images and messaging techniques that build trust and credibility through a mix of self-revelation, factual information, rapport, and appeals. While sharing similarities to traditional celebrities, social media influencers differ, in that they are relateable and imitable. While public health organisations clearly cannot replicate all these techniques, they can replicate the mix of techniques replacing some elements with alternatives, including role models or indeed influencers.
- Engineer—by engineer, we mean the practical application of scientific principles to the content value chain including the design, publication, and distribution. This involves optimising messaging, targeting, and amplification on an iterative basis, and where possible automating this process. In effect, this involves leveraging many of the same techniques used by enterprise marketers, bots, and spammers, including big data analytics, rule-based targeting, intensive automation, and optimisation of all elements of the content publishing process, including timing, repetition, hashtags, images, URLs, etc. Such tools may allow for public health organisations execute more effective counter messaging, but also increase their influence by being both visible and active. Successful optimisation requires an iterative approach of monitoring, analysis, planning, execution, and learning, often through controlled experimentation. The use of such tools and techniques is not without challenges. It requires specialist knowledge and skills, but also requires governance mechanisms to ensure that the use of such tools remains both ethical and compliant with relevant laws, regulations, and codes of conduct.
- Coordinate—nutrition and diet is not immune from the effects of globalisation and digitalisation. The public consume and engage with local and global influencers. This is clearly evident in the healthy diet discourse. There is significant commonality in public health guidelines worldwide, particularly in developed nations, and particularly across geo-political blocs such as the European Union. Notwithstanding this, most public health organisations and experts are organised and operate locally, despite social media being borderless. In much the same way that botmasters coordinate a swarm of accounts to amplify their message and present a particular viewpoint as being more popular or more widely accepted than it is in reality, by coordinating messaging and timing, nutrition and diet stakeholders can maximise their impact through mutual reinforcement on social media.

#### 5.1.1. Digital Health, Nutrition and Food Literacy

It is widely accepted that there is a need to promote sufficient skills in using information and communications technologies (ICTs) in general and for healthcare. As such, it is a not a significant stretch to suggest that there is a need for intervention in order to improve levels of health literacy with respect to nutrition, food, and diet information. Facing a deluge of content that includes both legitimate content and content that may be inaccurate, incomplete, manipulative, and spam, the general public need the knowledge and skills to make the right decisions for them based on understanding both the benefits and risks of a recommended action or behaviour. Sorensen et al. [102] define health literacy as: “knowledge, motivation and competencies to access, understand, appraise, and apply health information in order to make judgments and make decisions in everyday life concerning healthcare, disease prevention, and health promotion, to maintain or improve quality of life during the life course”.

This definition effectively seeks to integrate three literacies (functional, informative, and critical), as defined by Nutbeam [103] and two perspectives, that of public health and community health. In the context of nutrition, food, and diet literacy, two challenges arise. Firstly, while there is, in general terms, acceptance on the definition of health literacy, nutrition literacy and food literacy suffer from definitional ambiguity and lack widespread acceptance [104,105]. For example, in their review of nutrition and food literacy definitions, Krause [105] suggest that food literacy is a more viable term for health promotion interventions as it is more comprehensive while recognising the need for further harmonisation. The second challenge is that many of the functional, informative, and critical literacy skills assumed in these definitions have not been sufficiently adapted for Web 2.0. Similarly, there is a lack of accepted instruments for assessing digital health literacy. These skills include operational skills, navigation skills, information searching, evaluating reliability, determining relevance, adding self-generated content, and protecting privacy for both online health information and health care-related digital applications [106]. While some updated frameworks and scales have been developed for both digital health [106] and digital diet literacy, for example, the e-Healthy Diet Literacy (e-HDL) Scale [107], use, and acceptance are at a very nascent level.

Given these challenges, nutrition, food and diet stakeholders (including policy makers, nutritionists, dietitians, and their representative organisations, and the food industry) need to agree on what digital nutrition and food literacy is, what its relationship with literacy on diets and specifically special diets and supplements is, what the method for assessing the digital nutrition and food literacy levels of a population should be, and what intervention strategies are appropriate in order to increase literacy levels to an acceptable standard. As well as the general population, given the longer term risks that are associated with poor dietary behaviours in younger cohorts, we suggest that this will need to involve interventions in schools and universities.

#### 5.1.2. Platform Monitoring and Regulation

The typical focus of monitoring and regulation in public health communication are the health claims made by those marketing or promoting products and services and whether such claims are contrary to public health guidelines. Freedom of speech makes this more complicated when it comes to the general public or the promotion of special diet patterns and lifestyles. Our analysis suggests that, as well as countering misinformation and disinformation around nutrition and diet claims, public health communicators, nutritionists, dietitians, and their representative organisations need to influence greater monitoring, and failing that regulation, of platforms that disseminate such information. These include platforms for social networking and advertising.

The discussion with regards to monitoring and regulating social networking platforms, particularly in political and extremist contexts, is already ongoing. However, greater efforts need to be made by health, and in this case, nutrition and diet stakeholders. This may include lobbying or working with social networking, search, and other platforms to define strategies for identifying and labelling conflicts of interests (e.g., sponsored influencers), low quality nutrition and diet advice, misinformation, disinformation, and spam, and the accounts that dissemination them. In an ideal world, low quality information would be retracted and the accounts that disseminate these messages penalised somehow, including removal. The former assumes a pliant publisher. With content polluters, such as spammers and or bots, it is difficult to identify the controlling entity and there is little or no incentive to retract and comply with requests. In the event that a claim is retracted, the influence of the claim may still be felt long after, particularly if it has been shared extensively. Lewandowsky et al. [100] suggest that the most effective counter strategies for such messaging are (i) warnings at the time of the initial exposure, (ii) repetition of the retraction, and (iii) corrections that tell an alternative story that fills the coherence gap otherwise left by the retraction. While (ii) and (iii) assume a pliant publisher, (i) can be instituted at the platform level. Indeed, more recently, Twitter has labelled tweets with notices that warn users of sensitive content, for example, which may not be appropriate for all ages, or warnings on content that it considers counter to the public interest, for example for violating Twitter’s policy against abusive behaviour or misleading information. The latter has been famously used against Donald Trump a number of times [108]. Research does suggest that counter methods, such as warnings or counter information decrease the likelihood of sharing on Twitter [109]. As discussed, verified status may be a suitable proxy for trustworthiness and credibility on Twitter [82,83], while appropriate for accounts, verifying that every tweet may be infeasible and undesirable on the grounds of freedom speech.

With regards to advertising platforms, our research suggests that legitimate advertising platforms and advertisers play an indirect role in incentivising content polluters and spam in the nutrition and diet discourse, potentially inadvertently. Affiliate and programmatic advertisers are subject to moral hazards that are not observable and, if they are, not for some time after the payment for the advertising has been made [110]. This is particularly the case with programmatic advertising, where the profit opportunity, growth and scale, intense rivalry, and lack of transparency in the value chain serve to foment illegitimate and unethical practices, such as dynamic floor pricing, second price auctions, unethical agency volume bonus behaviours, inflated ad impressions, and data misuse [110]. It is increasing clear that in the absence of industry intervention, regulation is needed in order to address these issues to protect both consumers and advertisers. This includes greater monitoring by platforms, regulators, and advertisers.

## 6. Conclusions and Future Work

Public health education and the promotion of healthy diets is a key strategy for mitigating the adverse affects of being overweight and obesity. Increasingly, the general public source health, nutrition, and diet information on social media yet authoritative public health sources are lost in the healthy diet discourse by the noisy neighbours including those wishing to promote their products, services or worldview, and content polluters. This exploratory data analysis provides insights into both the actors and the content in the healthy diet discourse on Twitter, and by doing so can inform public health communication strategy and interventions to optimise communication and counter misinformation, disinformation, and other low quality messaging on social media.

This study is not without limitations; however, such limitations present significant opportunities for future research. First, while the explored dataset is extensive, it represents a limited portion of the discussion on nutrition, diet, and food generally, on social media. It is limited to specific set of keywords and hashtags. A healthy diet is only one part of the of the public health strategy to combat obesity, physical activity is also a critical component in mitigating the adverse impact of this disease. Future research should explore the wider discourse on food, physical exercise, and, indeed, other obesity interventions, for example, supplements. Second, our dataset is limited to one social networking site, Twitter, and one language, English. While society is more globalised and digital, there is a long-standing and established literature base on the impact of culture and other demographic factors on diet and health. Social networking sites and, in this case, Twitter, do not capture all segments of society and represent only one source of health information. Research is needed that compares influencers, topics, and different sources of healthy and diet information by demographic segment. Such research could be supplemented with primary qualitative research in order to understand the motivations and processing depth of health discourse engagement and secondary research using material shared on Twitter (reports, infographics, images, videos, etc.), but also major destination sites for these materials e.g., corporate websites, blogs, traditional media, and other social networks. Third, this study presented evidence of practices that are being used in order to deceive or manipulate the public e.g., bots. Further research is needed on the motivations, extent, and impact of such practices, including astroturfing, smoke screening, misdirection, and sock puppetry. Finally, this paper makes use of a number of analytical techniques, including descriptive analytics, content analytics, peak detection analysis, and network analysis. While there is a small but growing number of studies with large social media datasets to explore nutrition and diet topics while using machine learning techniques, there is a paucity of research using new techniques, such as deep learning, which can cater for the high dimensionality of Twitter data and efficiently model highly complex non-linear relationships between variables [111]. The use of such techniques may provide new and more generalisable insights.

## Figures and Tables

**Table 1:** Dataset Overview.

Message Type	No. of Tweets	% of Tweets
Original Tweets	545,543	45%
Retweets	581,913	48%
Replies	84,862	7%
Total	1,212,318	100%
	**No. of Users**	**% of Users**
Total	629,608	
Verified	7300	1%

**Table 2:** Number of Tweets by Country.

Full Dataset		Spam Users		Non-Spam Users		Verified Users	
Country	No. of Tweets	Country	No. of Tweets	Country	No. of Tweets	Country	No. of Tweets
United States	326,246	United States	90,078	United States	236,168	United States	6028
United Kingdom	119,805	United Kingdom	32,458	United Kingdom	87,347	United Kingdom	2667
India	48,819	India	8786	India	40,033	India	1249
Canada	33,485	Canada	7891	Canada	25,594	Canada	597
Australia	15,175	Belgium	6350	Australia	12,437	Australia	389
Malaysia	10,892	New Zealand	3124	Malaysia	10,654	Ireland	366
South Africa	9566	Australia	2738	South Africa	8580	South Africa	173
Nigeria	9218	Philippines	2042	Nigeria	8092	Italy	159
Philippines	8451	France	1585	Philippines	6409	Philippines	135
Belgium	7891	Russian Federation	1446	Ireland	5793	Switzerland	125
Ireland	7228	Ireland	1435	France	4866	Kenya	112
France	6451	Italy	1266	Spain	4554	Belgium	105
Spain	5447	Thailand	1151	Pakistan	4232	Nigeria	96
New Zealand	5230	Nigeria	1126	Germany	3776	Norway	83
Pakistan	4809	Mexico	1038	Indonesia	3758	United Arab Emirates	74
Germany	4780	Germany	1004	Kenya	2995	Spain	50
Indonesia	4533	South Africa	986	Italy	2990	France	48
Italy	4256	Spain	893	Mexico	2969	Pakistan	45
Mexico	4007	Indonesia	775	Saint Vincent and the Grenadines	2543	Germany	33
Kenya	3197	United Arab Emirates	657	United Arab Emirates	2349	Saudi Arabia	33

**Table 3:** Bot Score Summary.

Bot Score	Top 100 Active Users	Top 100 Visible Users
Very Low	2	82
Low	5	4
Medium	12	3
High	34	2
Very High	26	1
Suspended	21	8
Total	100	100

**Table 4:** Most Frequently Used Generators.

Generator	No. of Tweets
Twitter Clients	860,071
IFTTT	67,871
Facebook/Instagram	39,111
Hootsuite	36,796
Buffer	21,624
EdgeTheory	14,525
SocialOomph	12,042
WordPress.com	10,471
dlvr.it	7206
Bot Libre!	7205

**Table 5:** The 10 Most Influential Users in the *Health Diet* Network.

Account	PageRanks	Twitter Profile Description
southbeachdiet	0.00907580	Lose weight fast with our fully prepared delicious meals delivered right to your door!
DelilahVeronese	0.00132523	I’m nobody who are you? Do you feel like nobody too? Being a caregiver can be rewarding & a living hell. Don’t suffer alone.
SH_nutrition	0.00107434	Nutrition coach, cook & food writer based in Nottingham.Providing healthy eating advice & cookery lessons to individuals, groups & companies. Eat well feel well
realDonaldTrump	0.00100563	45th President of the United States of America
howudish	0.00086343	Dish discovery app that connects users to dishes fitting their nutritional lifestyle, and allows them to eat like pro athletes at local restaurants.
QunolOfficial	0.00059024	Qunol works tirelessly to provide the best quality CoQ10 and turmeric supplements on the market. Make the better choice and get Qunol CoQ10 or Turmeric today!
HealthWealthFi1	0.00043832	Always be positive. Think success, not failure. For exercise, develop a shorter, more convenient workout that you can use on unusually busy days.
NetMeds	0.00038329	Welcome to India’s most convenient pharmacy! A first-of-its-kind offering from the Dadha Group, the trusted name in pharma since 1914.
GMB	0.00035802	The UK’s most talked about breakfast television show. Weekdays from 6am on @ITV. Replies & content may be used on air. See http://itv.com/terms.
peta	0.00035744	Breaking animal news, #vegan recipes, rescues, & more from the largest animal rights organization in the world.

**Table 6:** Topic and Sub-topic Summary—Original Tweets.

	Original Tweets (N = 545,543)			
Topic	Frequency	Top 10 Subtopics	No. of Tweets	% of Tweets
Health	528,540	diet*	304,884	57.68%
		health/healthier/healthiest	32,188	6.09%
		life/live/lives/living	26,267	4.97%
		exercis*/fitness*/workout*	26,250	4.97%
		fat/fats	21,469	4.06%
		nutrition*	17,231	3.26%
		diabet*	10,973	2.08%
		disease*	6466	1.22%
		cancer*	5067	0.96%
		vitamin*	4561	0.86%
Ingest	496,143	diet*	304,884	61.45%
		eat/eating	93,517	18.85%
		food*	58,396	11.77%
		weight	55,292	11.14%
		fat/fats	21,469	4.33%
		meal*	13,730	2.77%
		veget*	12,202	2.46%
		fruit*	11,596	2.34%
		cook*	11,242	2.27%
		drink*	8056	1.62%

Note: * is a wildcard character.

**Table 7:** Summary of Prominent Sub-topics in Tweets by Spam Accounts.

	Spam Tweets (N = 151,183)			
Topic	Frequency	Top 10 Subtopics	No. of Tweets	% of Tweets
Health	147,721	diet*	74,501	50.43%
		health/healthier/healthiest	8799	5.96%
		fat/fats	8151	5.52%
		life/live/lives/living	6913	4.68%
		exercis*/fitness*/workout*	6144	4.16%
		nutrition*	3777	2.56%
		diabet*	2521	1.71%
		healing	1513	1.02%
		vital*	1513	1.02%
		pregnan*	1419	0.96%
Ingest	140,665	diet*	74,501	52.96%
		weight	24,326	17.29%
		eat/eating	23,350	16.60%
		food*	14,725	10.47%
		fat/fats	8151	5.79%
		cook*	4962	3.53%
		meal*	4131	2.94%
		veget*	3154	2.24%
		fruit*	2036	1.45%
		snack*	1963	1.40%

Note: * is a wildcard character.

**Table 8:** Summary of Prominent Sub-topics in Original Tweets by the All Accounts Excluding Verified and Spam Accounts.

	Original Tweets—No Spam (N = 394,360)			
Topic	Frequency	Top 10 Subtopics	No. of Tweets	% of Tweets
Health	380,197	diet*	230,379	60.59%
		health/healthier/healthiest	23,390	6.15%
		exercis*/fitness*/workout*	20,106	5.29%
		life/live/lives/living	19,355	5.09%
		nutrition*	13,454	3.54%
		fat/fats	13,350	3.51%
		diabet*	8451	2.22%
		disease*	5450	1.43%
		cancer*	3732	0.98%
		vitamin*	3490	0.92%
Ingest	354,383	diet*	230,379	65.01%
		eat/eating	70,167	19.80%
		food*	43,670	12.32%
		weight	30,992	8.75%
		fat/fats	13,350	3.77%
		meal*	9599	2.71%
		fruit*	9560	2.70%
		veget*	9048	2.55%
		drink*	6393	1.80%
		cook*	6280	1.77%

Note: * is a wildcard character.

**Table 9:** Summary of Prominent Sub-topics in Original Tweets by Verified Accounts.

	Original Tweets—Verified Users (N = 11,009)			
Topic	Frequency	Top 10 Subtopics	No. of Tweets	% of Tweets
Health	10,833	diet*	6994	64.56%
		health/healthier/healthiest	775	7.15%
		exercis*/fitness*/workout*	585	5.40%
		nutrition*	344	3.18%
		life/live/lives/living	523	4.83%
		disease*	207	1.91%
		cancer*	198	1.83%
		fat/fats	297	2.74%
		physical	142	1.31%
		diabet*	141	1.30%
Ingest	10,096	diet*	6994	69.27%
		food*	1486	14.72%
		eat/eating	1878	18.60%
		weight	734	7.27%
		veget*	294	2.91%
		fruit*	280	2.77%
		meal*	277	2.74%
		drink*	190	1.88%
		fat/fats	297	2.94%
		snack*	159	1.57%

Note: * is a wildcard character.

**Table 10:** Diets Mentions.

	**Original Tweets** **(N = 545,543)**		**Original Spam Tweets** **(N = 151,183)**	
**Diets**	**No. of Tweets**	**% of Tweets**	**No. of Tweets**	**% of Tweets**
High Protein and Low/No Carb	24,289	4.45%	9386	6.21%
Vegan, Vegetarian and Macrobiotic	15,743	2.89%	3392	2.24%
Gluten Free	832	0.15%	268	0.18%
Dairy Free	126	0.02%	23	0.02%
High Carb	68	0.01%	6	0.00%
Other	9172	1.68%	2541	1.68%
	**Original Tweets—No Spam** **(N = 394,360)**		**Verified Users** **(N = 11,009)**	
**Diets**	**No. of Tweets**	**% of Tweets**	**No. of Tweets**	**% of Tweets**
High Protein and Low/No Carb	14,893	3.78%	239	2.17%
Vegan, Vegetarian and Macrobiotic	12,351	3.13%	264	2.40%
Gluten Free	564	0.14%	6	0.05%
Dairy Free	103	0.03%	1	0.01%
High Carb	62	0.02%	4	0.04%
Other	6631	1.68%	214	1.94%

## Abbreviations

The following abbreviations are used in this manuscript:

API	Application Programming Interface
DDEO	Diabetes, Diet, Excercise and Obesity
eWOM	Electronic Word of Mouth
ICT	Information and Communication Technology
LDA	Latent Dirichlet Allocation
NHS	National Health Service
NLP	Natural Language Processing
TF-IDF	Term Frequency and Inverse Document Frequency
WEF	World Economic Forum
WHO	World Health Organization
WOM	Word of Mouth
